# UK Parliament’s antimicrobial resistance inquiry: translating evidence into crisis-resilient action

**DOI:** 10.1093/jacamr/dlaf218

**Published:** 2025-11-19

**Authors:** Rasha Abdelsalam Elshenawy

**Affiliations:** Department of Medicine, School of Health, Medicine and Life Sciences, University of Hertfordshire, Hatfield, UK

## Abstract

Parliamentary inquiries play a crucial role in bridging scientific evidence and health policy. In response to the United Kingdom (UK) Parliament’s Public Accounts Committee inquiry on antimicrobial resistance (AMR), this Viewpoint presents a ‘crisis-resilient antimicrobial stewardship’ model designed to sustain effectiveness during emergencies while strengthening routine performance. The model is organized around five interconnected pillars: digital innovation and diagnostics, One Health and environmental governance, workforce resilience and training, surveillance and data integration, and governance and policy alignment. Positioned within the global policy context of the World Health Organization (WHO), the European Union (EU), and the Sustainable Development Goals, it embeds equity, sustainability, and accountability. Aligning national reforms with international frameworks can enhance the UK’s contribution to global AMR resilience and the 2030 Agenda.

## Introduction

The United Kingdom (UK) Parliament’s Public Accounts Committee inquiry on antimicrobial resistance (AMR) in 2025 represents a pivotal step in embedding science within health policy.^1^ The inquiry validated long-standing academic concerns regarding missed diagnostic targets, workforce shortages, and fragmented governance.^2^ These challenges align with global frameworks such as the WHO Global Action Plan on AMR (2015), the EU One Health Action Plan (2017) and its associated Roadmap on One Health AMR governance, and the Quadripartite One Health Joint Plan of Action (2022–26).^3-5^ The Committee’s findings provide an opportunity to operationalize these international objectives through a UK-led model of crisis-resilient antimicrobial stewardship (AMS) that enhances preparedness, accountability and cross-sector collaboration.^2^ These reforms also align with WHO’s pandemic-preparedness agenda, ensuring AMR remains central to global health security frameworks. The parliamentary assessment illustrates the growing integration of science and policy, where academic research directly informs parliamentary scrutiny and evidence-based governance.^6^ Written evidence submissions form part of a wider knowledge-mobilization ecosystem that translates empirical findings into institutional reform. This approach reflects the UK Health Security Agency’s (UKHSA) Science Strategy (2023), which prioritizes research impact assessment, academic partnerships, and knowledge translation as key enablers of AMR preparedness and public health resilience.^2,7^ Collectively, these developments demonstrate the UK’s systemic commitment to embedding evidence-informed governance within national and global AMR policy.

## From research evidence to parliamentary validation

Written evidence to the Committee highlighted how COVID-19 exposed weaknesses in AMS, particularly the overuse of antibiotics during viral infections.^7^ The Committee confirmed these findings, noting increased precautionary prescribing alongside persistent gaps in diagnostics and antibiotic use.^8–14^ It also identified that one in five antibiotic prescriptions remains inappropriate, reinforcing the need for improved diagnostics and digital oversight.^13,14^ Figure S1 (available as Supplementary data at *JAC-AMR* Online) summarizes the evolution from pre-pandemic manual systems to digitally adaptive stewardship frameworks. The Committee’s recommendations on sustainable AMS practice, governance, surveillance integration and workforce development directly align with the crisis-resilient principles outlined in the submission ensuring evidence translates into actionable reform.^2^

## Crisis-resilient AMS: the five-pillar framework

**Figure 1.**
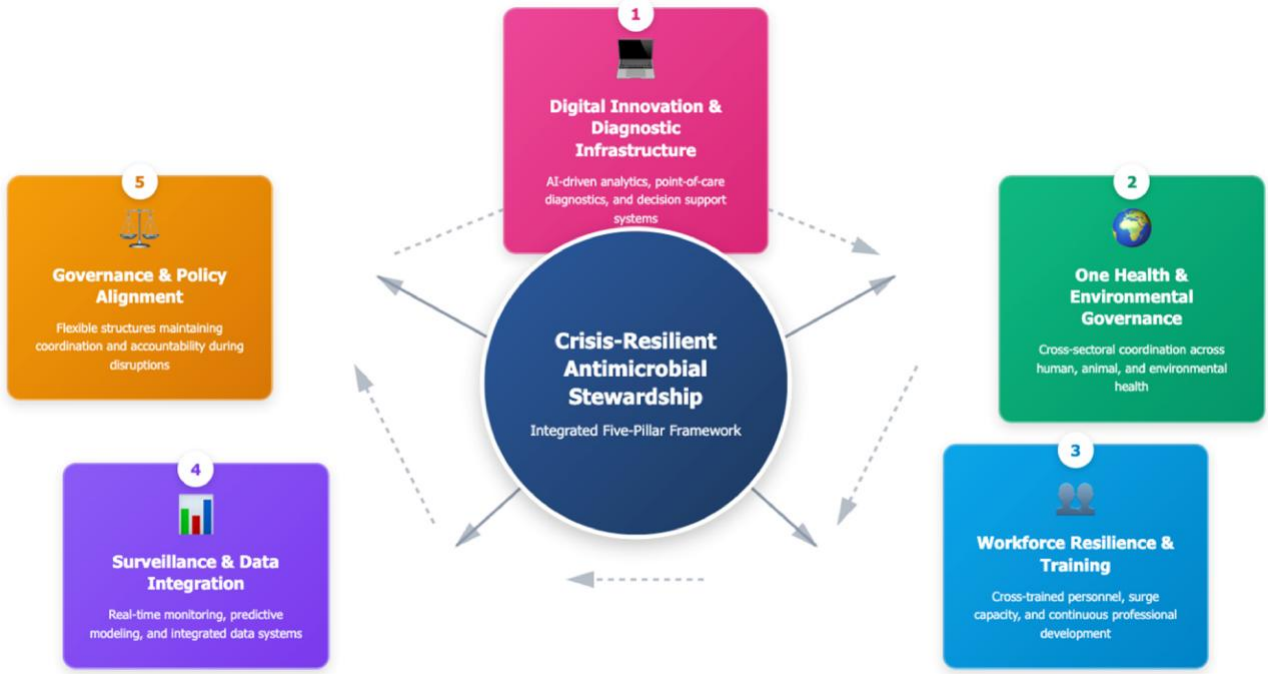
Conceptual framework for crisis-resilient antimicrobial stewardship.

Crisis-resilient AMS is a governance model that maintains and adapts stewardship effectiveness during both stable and emergency conditions. It comprises five interconnected dimensions: (i) adaptive workforce capacity; (ii) robust diagnostic infrastructure; (iii) real-time cross-sector data systems; (iv) flexible governance ensuring coordination and accountability; and (v) digital innovation supporting predictive analytics and clinical decision support. Together, these elements transform AMS from a compliance process into a dynamic, learning-oriented system that safeguards antimicrobial effectiveness and strengthens preparedness for future health crises. The interconnections between these components are illustrated in Figure 1.^15,16^

### Pillar 1: digital innovation and diagnostic infrastructure

Digitalization and diagnostic capacity form the foundation of crisis-resilient stewardship. Parliamentary evidence highlighted gaps in diagnostic implementation and data integration. Research during the coronavirus disease 2019 (COVID-19) pandemic demonstrated that electronic dashboards displaying antimicrobial use and resistance improved oversight and feedback.^8,9,10,13^ Aligning such tools with the WHO Digital Health for AMR initiative and the EU One Health Action Plan enhances global interoperability, knowledge exchange, and mobilization.^17^ Addressing the digital divide through affordable, locally hosted, interoperable systems is essential to ensure equitable diagnostic access in all settings.

### Pillar 2: one health and environmental governance

Environmental neglect remains a major driver of AMR. The UK Parliament reported increasing wastewater contamination, reinforcing the need for stronger regulation and coordinated surveillance.^2^ Implementing extended producer-responsibility schemes would ensure pharmaceutical manufacturers manage antimicrobial waste responsibly. Wastewater-based epidemiology could enable early detection of resistance genes in sewage and hospital effluents, linking environmental and public-health monitoring. Regulatory controls on veterinary and agricultural antimicrobial use, coordinated by the Department for Environment, Food and Rural Affairs (DEFRA), NHS England and UKHSA, would align national reforms with WHO and EU guidance on sustainable One Health AMR governance.^7,18,19^

### Pillar 3: workforce resilience and training

A skilled and adaptable workforce is central to effective stewardship.^9^ The Public Accounts Committee identified a 20% deficit in consultant microbiologists and uneven AMS expertize across the country.^2^ Cross-trained, simulation-ready professionals and expanded Pharmacy First AMS education are priorities. AMS competencies should be embedded in continuing professional development and inter-professional training in line with the UK AMR Action Plan 2024–29.^7,11,15^ Addressing geographic maldistribution is equally important. Incentivized placements, remote mentorship, and decentralized education programmes can strengthen capacity in underserved areas, ensuring equitable workforce distribution and national resilience.

### Pillar 4: surveillance and data integration

Integrating surveillance across human, animal, and environmental sectors transforms stewardship from reactive to preventive. Artificial intelligence (AI) and machine-learning applications can enhance early detection and response. Alignment with the WHO Global Antimicrobial Resistance and Use Surveillance System (GLASS) and the EU EARS-Net ensures standardized data quality and comparability.^20,21^ Incorporating information from rural laboratories, community pharmacies, and veterinary facilities promotes inclusion and comprehensive monitoring. Linking wastewater and clinical data through national AMR dashboards, similar to the ECDC European Surveillance System (TESSy) system, can identify emerging resistance hotspots and guide timely interventions.^10,21^

### Pillar 5: governance and policy alignment

Governance underpins the previous pillars. Parliamentary recommendations for improved transparency and accountability should be operationalized through coherent legislative and performance frameworks. Integrating AMR indicators into NHS performance dashboards, linking funding to measurable outcomes, and ensuring ongoing parliamentary oversight can sustain accountability beyond electoral cycles.^7,10^ Embedding AMR resilience indicators in the UK National Action Plan (2024–29) will ensure that stewardship progress is evidence-based and strategically aligned with long-term health-security goals.^19,22^ Embedding AMR oversight within national crisis-response mechanisms will also ensure continuity of stewardship during future health emergencies.

## From framework to action: implementing crisis-resilient stewardship

Effective implementation of the crisis-resilient stewardship framework requires coordinated leadership from the Department of Health and Social Care (DHSC), NHS England, DEFRA and the UKHSA.^3,5^ Short-term priorities (0–12 months) include establishing AMR coordination boards and embedding stewardship metrics within NHS performance frameworks. Medium-term actions (1–3 years) focus on expanding digital stewardship platforms, scaling diagnostic access, and piloting wastewater-based surveillance. Long-term goals (3–5 years) involve legislating extended producer-responsibility schemes, integrating national AMR data systems, and institutionalizing AMR resilience indicators. Detailed timelines and responsible actors are outlined in Table S1.

## Global adaptability and United Nations sustainable development goals alignment

Although this framework is grounded in the UK context, its principles are globally transferable. In low- and middle-income countries, digital systems can be implemented using open-source, low-bandwidth platforms; regional surveillance hubs can strengthen One Health data exchange; and workforce capacity can be enhanced through South–South collaboration and WHO technical assistance.^9,18,19^ The framework aligns with Sustainable Development Goals (SDGs) 3.d (health security), 6.3 (water quality), 12.4 (waste management), and 17.16 (global partnerships). Each pillar’s alignment with specific SDG targets is mapped in Table S2, illustrating how crisis-resilient stewardship supports the 2030 Agenda for Sustainable Development.^10,22^

## Future directions

This Viewpoint focuses primarily on UK governance and may require adaptation for other settings with different institutional capacities. The proposed framework requires empirical validation through implementation studies. Future research should include comparative and economic evaluations of AMS interventions, mixed-methods studies of stakeholder perspectives, and longitudinal research on stewardship performance during both stable and crisis periods. Implementation science is essential to identify practical barriers and enablers to embedding resilience in stewardship systems.

### Conclusion

The UK Parliament’s AMR inquiry highlights the vital role of evidence-informed governance and knowledge mobilization in translating research into sustainable stewardship reform that saves lives. Building on written evidence submitted to the Committee, this Viewpoint demonstrates how translating scientific evidence into policy can strengthen both national strategy and global preparedness to fight AMR. This crisis-resilient AMS framework, structured around five interconnected pillars, provides a scalable model for embedding adaptability, accountability, and equity within stewardship systems. Aligned with WHO GLASS, the EU One Health Action Plan, and the United Nations SDGs, it supports collective progress towards resilient and sustainable AMR governance. Timely adoption of this framework would enhance global collaboration and accelerate the translation of evidence into life-saving policies to combat AMR.

## Supplementary Material

dlaf218_Supplementary_Data

## Supplementary data

Figure S1 and Tables S1 and S2 are available as Supplementary data at *JAC-AMR* Online.
