# Supplementary material for: UK Parliament’s antimicrobial resistance inquiry: translating evidence into crisis-resilient action
Source: JAC Antimicrob Resist. 2025 Nov 19;7(6):dlaf218. doi: 10.1093/jacamr/dlaf218 (PMC12628752; doi:10.1093/jacamr/dlaf218)
Supplement: dlaf218_Supplementary_Data [file dlaf218_supplementary_data.zip › Supplementary_Table_1_AMR_Implementation_Roadmap.docx]

## Supplementary Table 1. Implementation roadmap for operationalising crisis-resilient antimicrobial stewardship within existing UK AMR governance frameworks

| Pillar / Focus Area | Primary Responsible Actors | Key Implementation Actions | Timeline | Enabling Conditions / Integration Points |
| --- | --- | --- | --- | --- |
| Pillar 1: Digital Innovation and Diagnostic Infrastructure | UKHSA, NHS England, DHSC, NIHR AI Lab | Launch Diagnostics Accelerator; integrate stewardship dashboards into NHS EHR; expand tele-stewardship and remote prescribing audits | Short-term (0–12 months) and Medium-term (1–3 years) | Funding from DHSC Innovation Fund; NHS Digital Strategy; alignment with WHO Digital Health for AMR and EU One Health Action Plan |
| Pillar 2: One Health and Environmental Governance | DEFRA, UKHSA, Environment Agency, NHS England | Implement extended producer responsibility (EPR) schemes for pharmaceutical waste; establish wastewater-based epidemiology (WBE) surveillance; strengthen veterinary antimicrobial regulation | Medium-term (1–3 years) and Long-term (3–5 years) | Environmental Act framework; inter-agency coordination; alignment with WHO–FAO–WOAH–UNEP Joint Plan of Action (2022–2026) |
| Pillar 3: Workforce Resilience and Training | DHSC, Health Education England (HEE), NHS Trusts, Royal Pharmaceutical Society | Develop national AMS competency framework; expand Pharmacy First training; pilot remote mentorship and simulation-based learning for underserved areas | Short-term (0–12 months) to Medium-term (1–3 years) | Integration with UK AMR Action Plan (2024–2029); CPD accreditation; NHS workforce expansion programme |
| Pillar 4: Surveillance and Data Integration | UKHSA, NHS England, DEFRA, ECDC collaboration | Link GLASS and EARS-Net indicators to NHS dashboards; integrate environmental data via TESSy platform; develop AI-based early-warning analytics | Medium-term (1–3 years) and Long-term (3–5 years) | Data Strategy 2023; interoperability standards; cross-sector data-sharing agreements |
| Pillar 5: Governance and Policy Alignment | DHSC, Cabinet Office, UK Parliament Select Committees, devolved administrations | Establish cross-sector AMR Stewardship Board; legislate AMR accountability metrics; embed stewardship KPIs into NHS performance framework | Short-term (0–12 months) to Long-term (3–5 years) | NHS Long Term Plan; National AMR Action Plan (2024–2029); parliamentary oversight mechanisms |
