# Supplementary material for: UK Parliament’s antimicrobial resistance inquiry: translating evidence into crisis-resilient action
Source: JAC Antimicrob Resist. 2025 Nov 19;7(6):dlaf218. doi: 10.1093/jacamr/dlaf218 (PMC12628752; doi:10.1093/jacamr/dlaf218)
Supplement: dlaf218_Supplementary_Data [file dlaf218_supplementary_data.zip › Supplementary_Table_2_SDG_Alignment.docx]

## Supplementary Table 2. Alignment of Crisis-Resilient Stewardship Pillars with United Nations (UN) Sustainable Development Goals (SDGs)

| Pillar | Relevant SDG Targets | Contribution to 2030 Agenda |
| --- | --- | --- |
| Pillar 1: Digital Innovation and Diagnostic Infrastructure | SDG 3.d, SDG 17.16 | Enhances early warning, digital capacity, and global data partnerships for AMR surveillance. |
| Pillar 2: One Health and Environmental Governance | SDG 6.3, SDG 12.4 | Reduces pollution through antimicrobial waste control, wastewater monitoring, and sustainable practices. |
| Pillar 3: Workforce Resilience and Training | SDG 3.c, SDG 17.9 | Strengthens health workforce capacity and international cooperation for sustainable training systems. |
| Pillar 4: Surveillance and Data Integration | SDG 3.d, SDG 17.18 | Promotes integrated, high-quality data systems for risk detection and cross-sector accountability. |
| Pillar 5: Governance and Policy Alignment | SDG 16.6, SDG 17.16 | Reinforces transparent, inclusive, and globally aligned AMR governance mechanisms. |
