# Supplementary figures and images for: UK Parliament’s antimicrobial resistance inquiry: translating evidence into crisis-resilient action
Source: JAC Antimicrob Resist. 2025 Nov 19;7(6):dlaf218. doi: 10.1093/jacamr/dlaf218 (PMC12628752; doi:10.1093/jacamr/dlaf218)

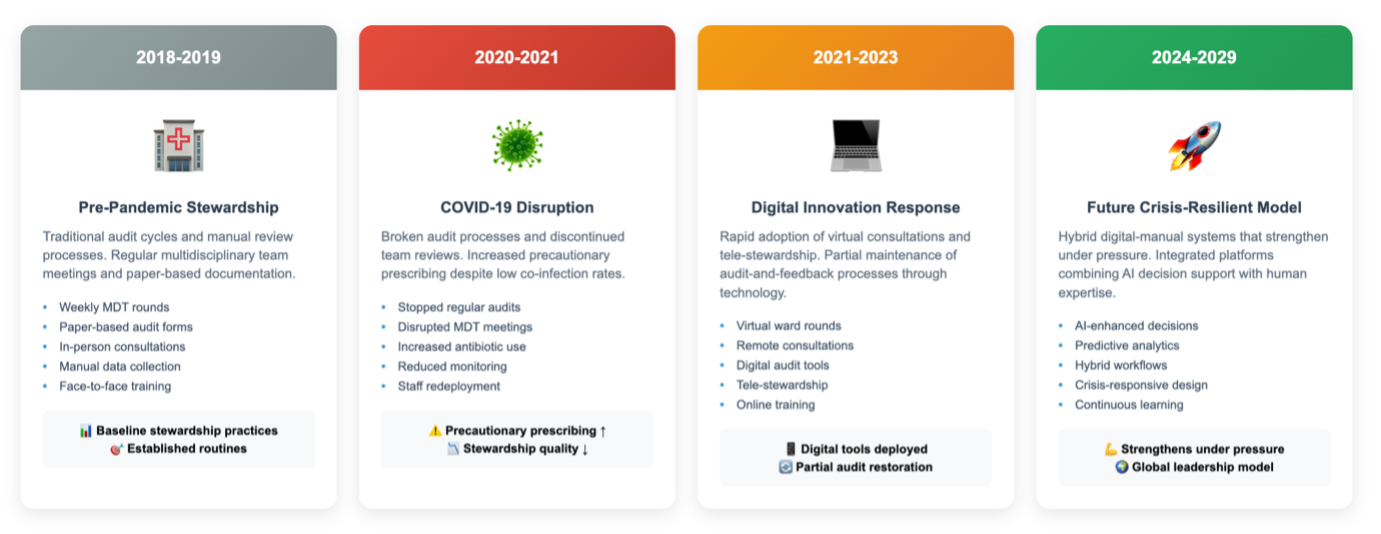

Supplement: dlaf218_Supplementary_Data [file dlaf218_supplementary_data.zip › Supplementary Figure 1.png]
